# Supplementary material for: Exposure of Von Willebrand Factor Cleavage Site in A1A2A3-Fragment under Extreme Hydrodynamic Shear
Source: Polymers (Basel). 2021 Nov 12;13(22):3912. doi: 10.3390/polym13223912 (PMC8625202; doi:10.3390/polym13223912)
Supplement: Supplementary file 1 [file polymers-13-03912-s001.zip › polymers-1448365-supplementary.pdf]

Supporting Information. Exposure of von Willebrand factor cleavage site in A1A2A3-fragment under extreme hydrodynamic shear.

O. Langguin-Cattoën,[†,‡] E. Laborie,[†,‡] D. O. Yurkova,[¶] S. Melchionna,[§,∥] P. Derreumaux,[†,‡] A. V. Belyaev,[*,¶] and F. Sterpone[*,†,‡]

†*CNRS, Université de Paris, UPR 9080, Laboratoire de Biochimie Théorique, 13 rue Pierre et Marie Curie, F-75005, Paris, France*

‡*Institut de Biologie Physico-Chimique-Fondation Edmond de Rothschild, PSL Research University, Paris, France*

¶*Lomonosov Moscow State University, Faculty of Physics, 119991 Moscow, Russia*

§*ISC-CNR, Dipartimento di Fisica, Università Sapienza, P.le A. Moro 5, 00185, Rome, Italy*

∥*Lexma Technology 1337 Massachusetts Avenue, Arlington, MA 02476, USA*

E-mail: aleksey_belyaev@yahoo.com; fabio.sterpone@ibpc.fr

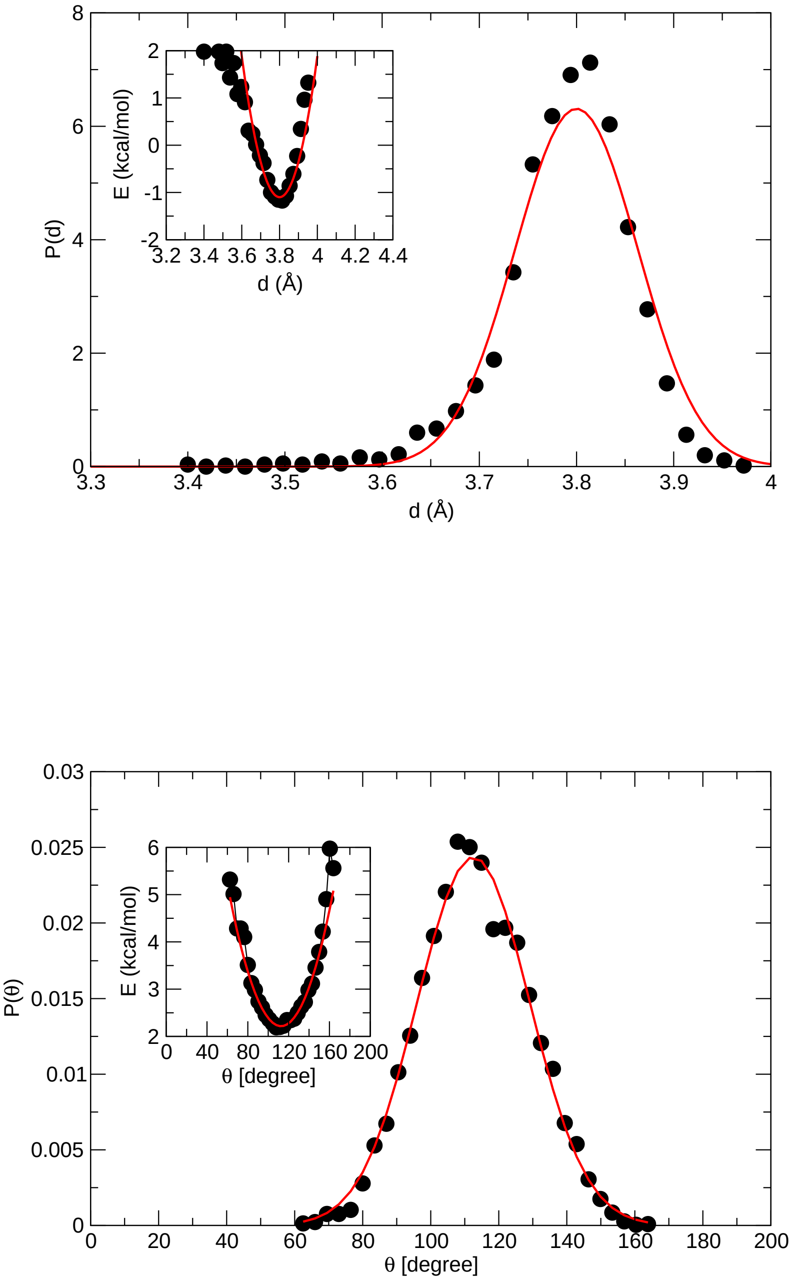

Figure S1: **Coarse-grain potential for inter-domains linker.** Probability distribution of the C_α-C_α distances (top) of the angle θ between three consecutive C_αs (bottom) extracted from the simulation of the L_{A1A2} linker based on the full flexible OPEP model.

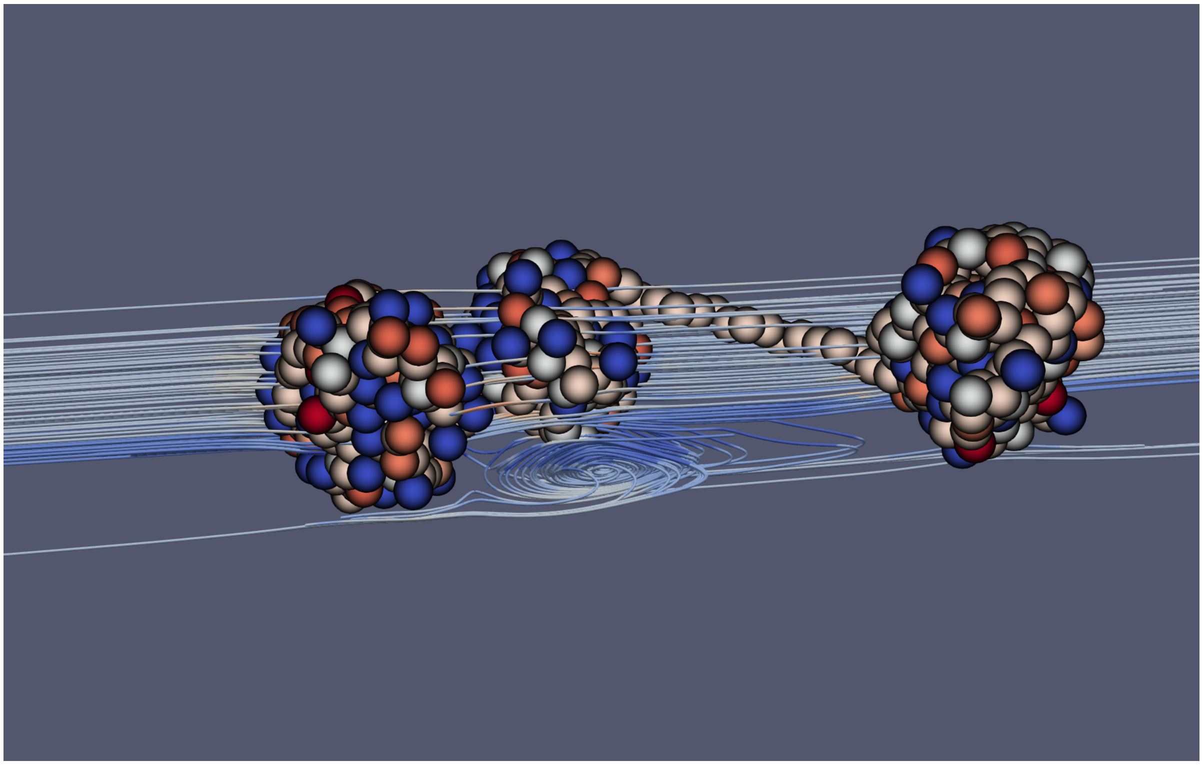

Figure S2: **A1A2A3 under shear.** Representation of the effect of the coupling between protein and fluid motion, the streamlines of the velocity field show a complex pattern in the proximity of the molecular construct, deviating from the linear geometry expected in an unperturbed Couette flow.

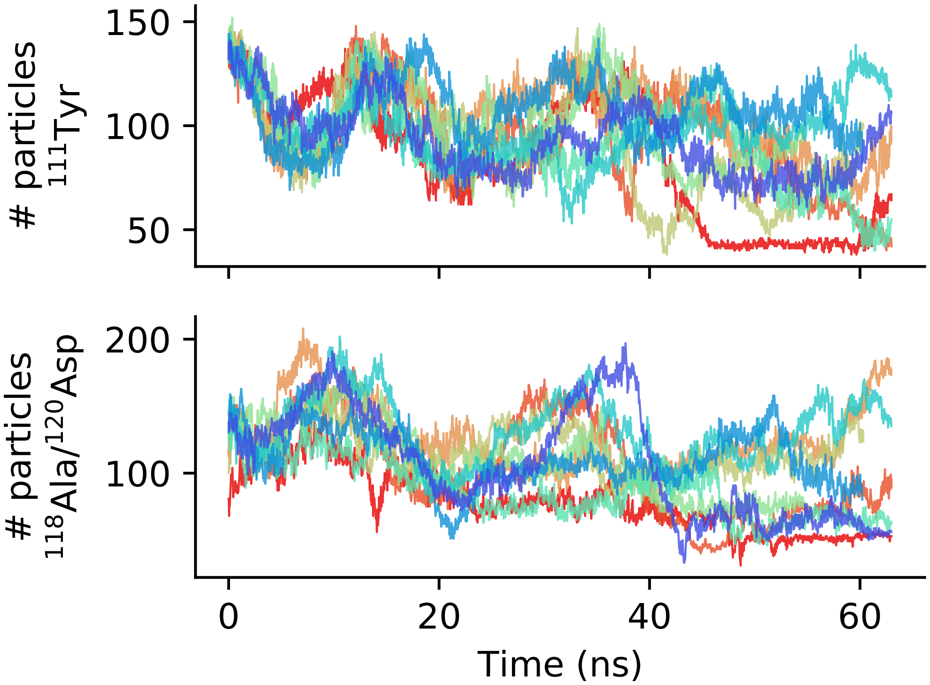

Figure S3: **Exposure of the cleavage and the recognition sites of A2 domain**. Time evolution of the coordination number of ^{111}Tyr (top panels) and ^{118}Ala and ^{120}Asp residues (bottom panels) for the A2 domain unfolding under shear flow.

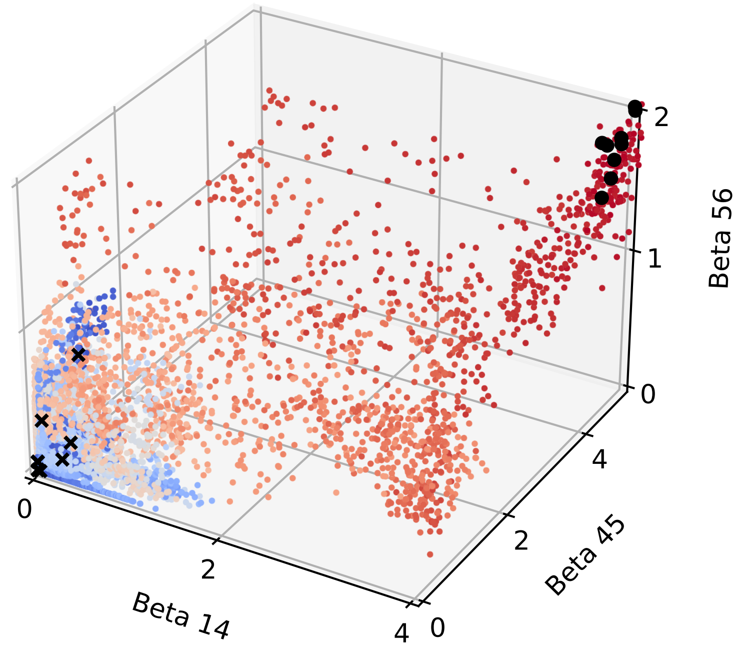

Figure S4: **Unfolding path of the A2 domain under shear.** The trajectories are projected on the 3-dimensional CV space of the number of contacts between beta strands 1 and 4, 4 and 5, 5 and 6. Black dots and crosses denote the beginning and ending of the trajectories, respectively. Trajectories are colored from red (beginning) to blue (ending).

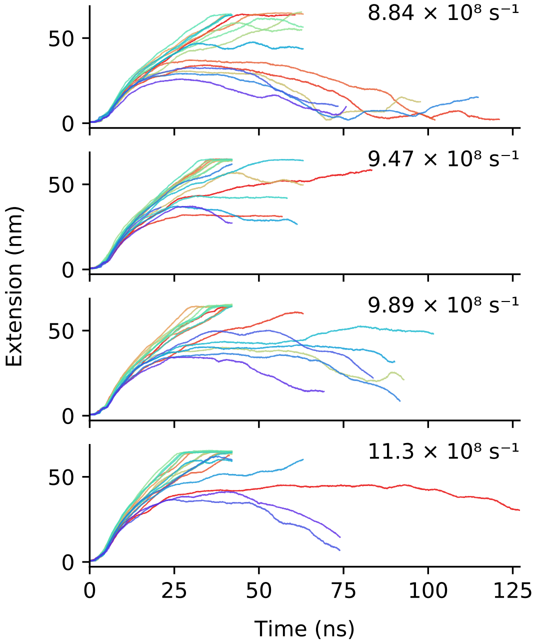

Figure S5: **Extension of the A2 domain within the A1A2A3 construct under shear**. Time evolution of the end-to-end distance of the A2 domain during the shear-induced unfolding of A1A2A3 construct. In the four panels we report data for several shear rates.

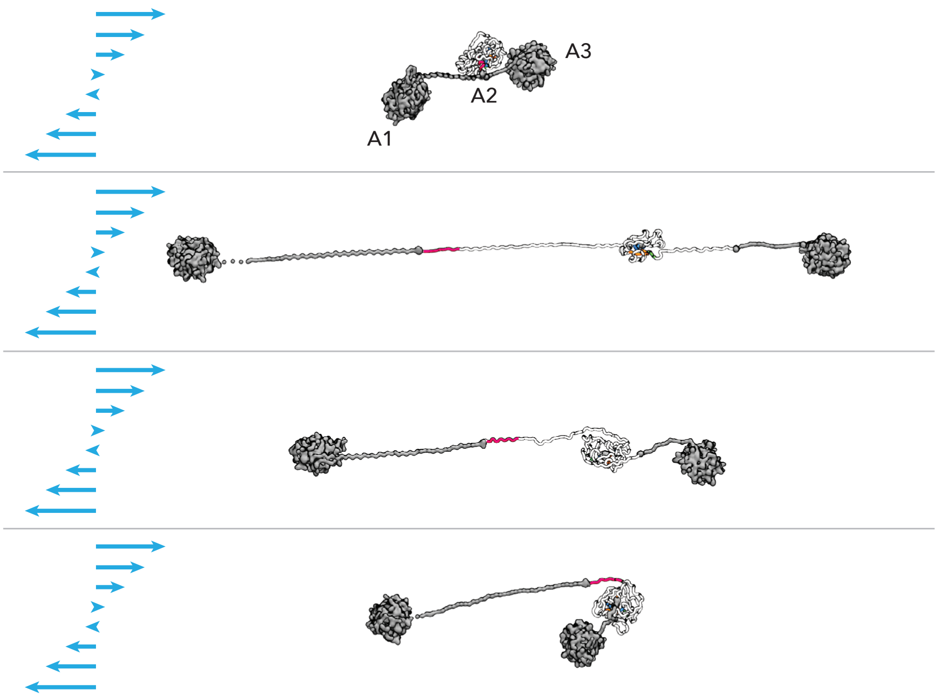

Figure S6: **A1A2A3 under shear.** Partial unfolding and refolding of the A2 domain under the action of the elongation and rotational components of the shear flow field.

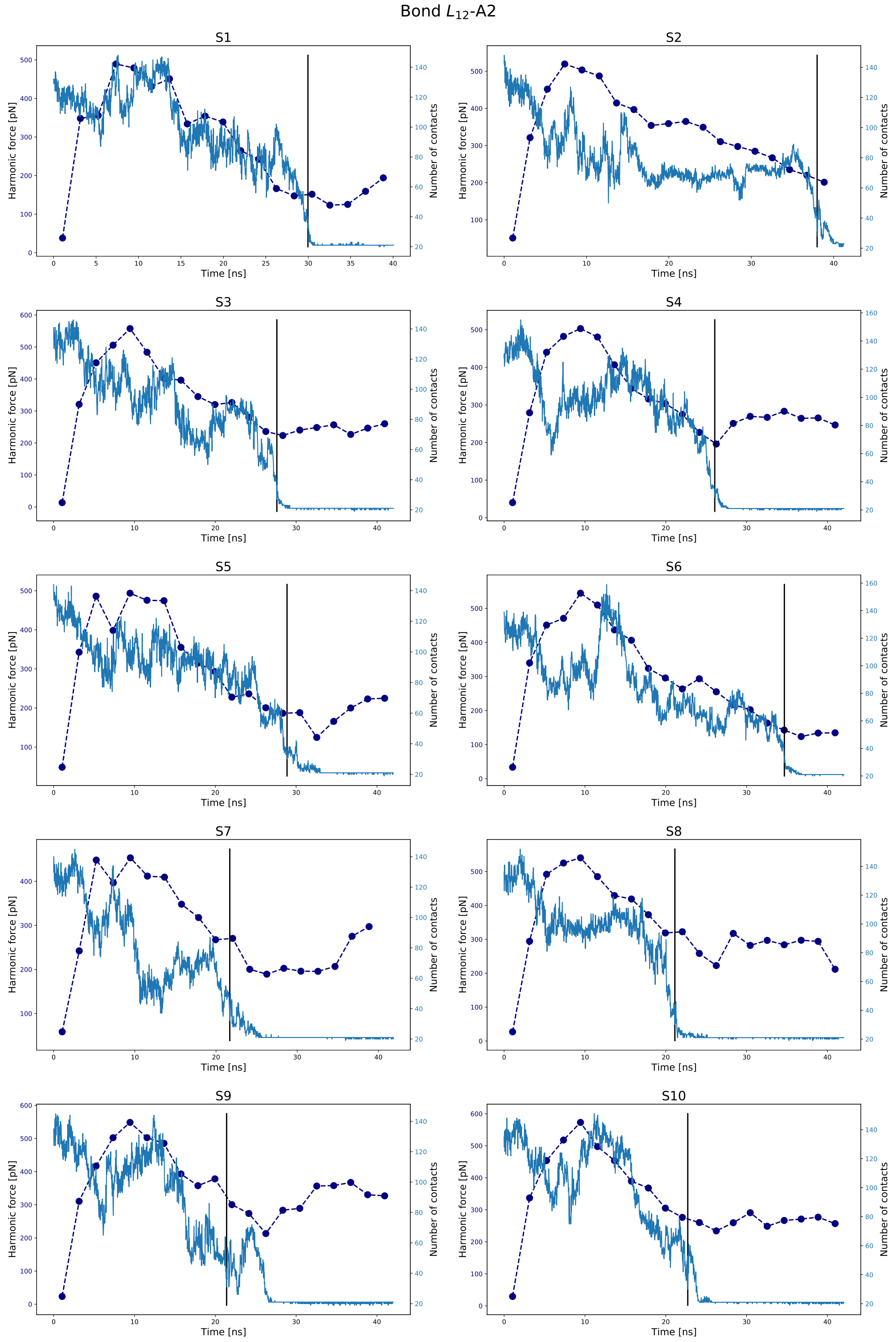

Figure S7: **Effective load force on A2 terminals.** Effective loading force generated by the shear flow at the two terminals of the A2 domain linked to A1. The vertical black line corresponds to the exposure time of the cleavage site. The force is estimate using a block average of 2.1 ns. The shear value is $\dot{\gamma} = 1.13 \cdot 10^9\, s^{-1}$.

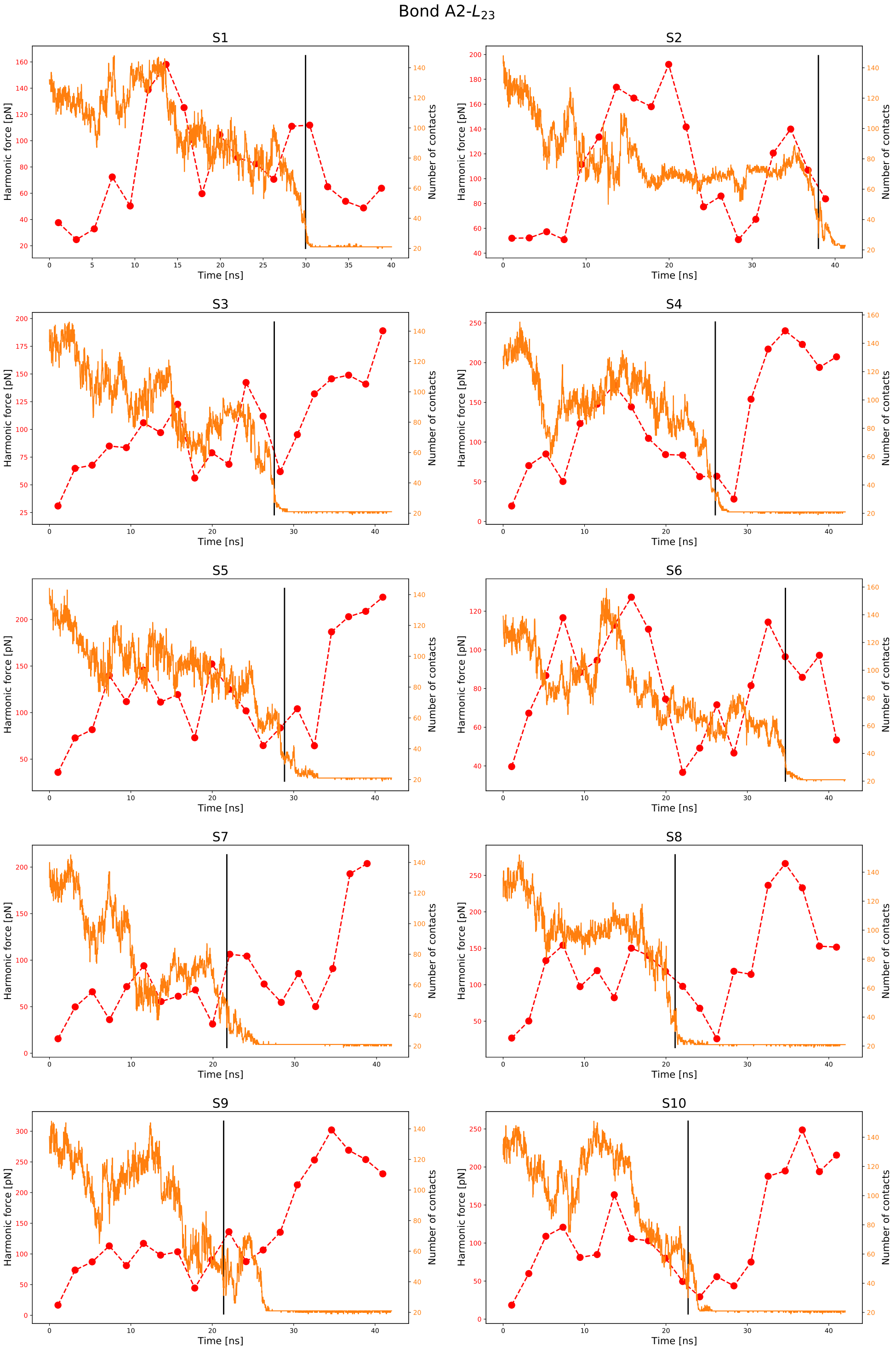

Figure S8: **Effective load force on A2 terminals.** Effective loading force generated by the shear flow at the two terminals of the A2 domain linked to A3 (orange). The vertical black line corresponds to the exposure time of the cleavage site. The force is estimate using a block average of 2.1 ns. The shear value is $\dot{\gamma} = 1.13 \cdot 10^9\ s^{-1}$.

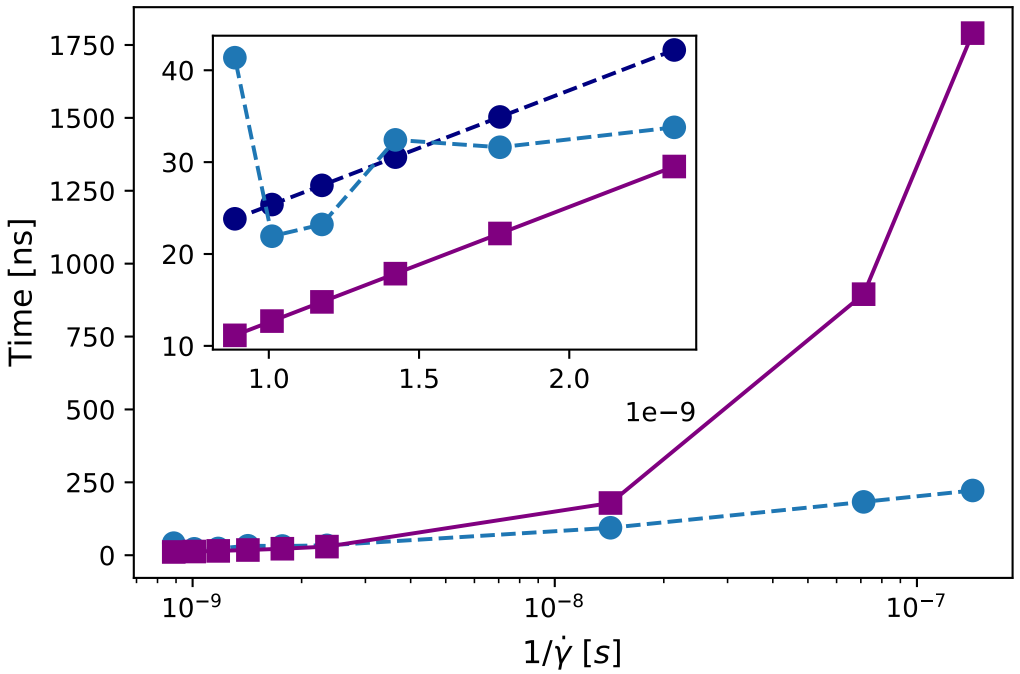

Figure S9: **A1A2A3 rotation under shear.** Characteristic rotational time of A1 domain with respect to A2 (blue) obtained by averaging independent runs at different shear rate and compared to the rotational period associated to a Couette flow ($T_r = 4\pi/\dot{\gamma}$) (purple). A linear fit is performed (dark blue) in the regime ($\dot{\gamma} \sim 10^9 s^{-1}$) where the rotational dynamics follows the theoretical prediction.

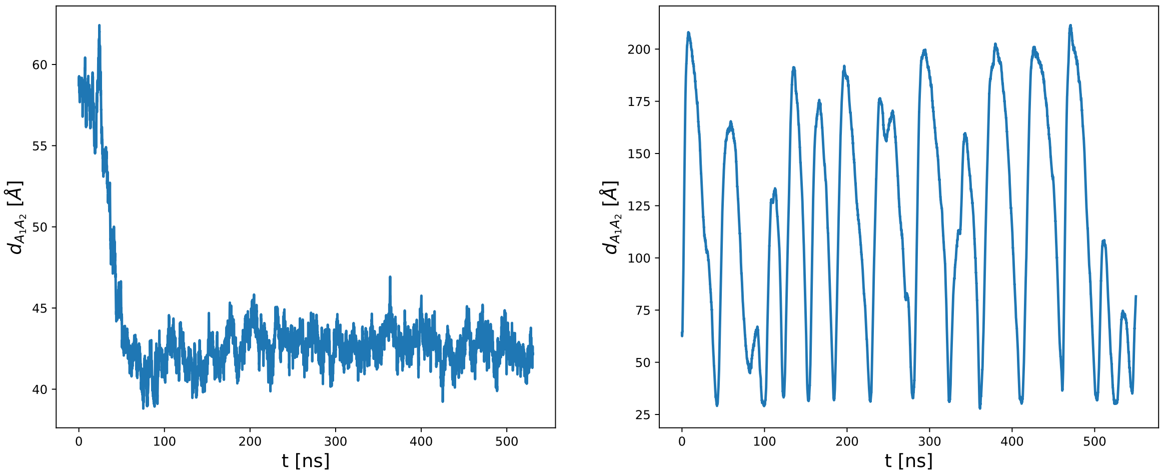

Figure S10: **A1A2A3 collapse/extension dynamics.** Time evolution of the A1A2 inter-domain distance for low (right) and high (left) shear rates, respectively $1.4 \times \dot{\gamma} \sim 10^5 s^{-1}$ and $8.5 \times \dot{\gamma} \sim 10^8 s^{-1}$.
